# Risk factors and the resistance mechanisms involved in *Pseudomonas aeruginosa* mutation in critically ill patients

**DOI:** 10.1186/s40560-019-0390-4

**Published:** 2019-07-19

**Authors:** Stéphanie Druge, Stéphanie Ruiz, Fanny Vardon-Bounes, Marion Grare, François Labaste, Thierry Seguin, Olivier Fourcade, Vincent Minville, Jean-Marie Conil, Bernard Georges

**Affiliations:** 1 Service de Réanimation Polyvalente, CHU Rangueil, 1 Avenue Jean Poulhès, Pôle d’Anesthésie et Réanimation, TSA 50032, 31059 Toulouse Cedex 9, France; 2 Laboratoire de Bactériologie et Hygiène, Institut Fédératif de Biologie, 330 Avenue de Grande Bretagne, TSA 40031, 31059 Toulouse Cedex 9, France; 3 Department of Anesthesiology and Intensive Care Units, University Hospital of Toulouse, 31059 Toulouse Cedex 9, France

**Keywords:** *Pseudomonas aeruginosa*, Mutation, Antibiotic, Resistance mechanism, ICU (intensive care unit), Segmentation tree

## Abstract

**Background:**

The objective of this study was to determine the main risk factors of *Pseudomonas aeruginosa* mutation as well as the mechanisms of acquired resistance.

**Methods:**

We conducted a 2-year prospective study in patients who were carriers of a *Pseudomonas aeruginosa* strain and who had been admitted to a medical/surgical ICU.

**Results:**

Of the 153 patients who were included, 34 had a mutation in their strain. In a multivariate analysis, a duration of ventilation > 24 days was a risk factor for mutation (risk ratio 4.29; CI 95% 1.94–9.49) while initial resistance was a protective factor (RR 0.36; CI 95% 0.18–0.71). In a univariate analysis, exposure of *P. aeruginosa* to ceftazidime was associated with an over-production of AmpC cephalosporinase and exposure to meropenem was associated with impermeability. A segmentation method based on the duration of ventilation (> 24 days), initial resistance, and exposure of strains to ceftazidime made it possible to predict at 83% the occurrence of mutation.

**Conclusion:**

The duration of ventilation and the presence of resistance as soon as *P. aeruginosa* is identified are predictive factors of mutation in ICU patients.

## Background

*Pseudomonas aeruginosa* is the primary microorganism responsible for nosocomial infections in the ICU [1, 2]. The prognosis for these infections is very poor with a mean mortality of 30% [3–5] and can be as high as 50 to 60% [6]. The prevalence of *P. aeruginosa* infections and the rates of antipyocyanic antibiotic resistance are on a rise internationally [7, 8].

*P. aeruginosa* is characterized by a high level of natural resistance to antibiotics and by its capacity to acquire new resistance mechanisms by chromosomal mutations or horizontal transmission of genetic materials [9], with the resulting risk of an unadapted antibiotic therapy [5, 10]. Mutation can also occur during treatment and generate therapeutic failure. According to studies, 6 to 53% of the *P. aeruginosa* infections that are treated will become resistant to one or more antipyocyanic antibiotics [11, 12]. This acquisition of resistance is associated with an increase in mortality, the duration of stay, and cost [1, 13, 14].

Exposure to antibiotics is one of the primary risk factors for the acquisition of resistance that is studied, notably to the fluoroquinolones and carbapenems. Policies that restrict the use of certain antibiotics including the fluoroquinolones have shown a decrease in the rate of resistance to these antibiotics as well as to the other classes [15]. Therefore, it would appear that it is important to address the risk factors for mutation and the resistance mechanisms induced by the various antipyocyanic antibiotics, in order to limit the use of those that contribute the most to resistance, in keeping with the experts’ recommendations [16].

Therefore, we conducted a study for which the main objective was to identify risk factors for the mutation of *Pseudomonas aeruginosa* strains in patients hospitalized on our unit.

The secondary objectives were to identify the resistance mechanisms induced by these antibiotics and to identify any correlation between the main antipyocyanic antibiotics prescribed and the different resistance mechanisms acquired by *Pseudomonas aeruginosa.*

## Methods

We conducted a 2-year open prospective observational study in the Medical/Surgical ICU of Rangueil tertiary hospital, Toulouse.

This project was approved by the Toulouse Hospital Ethics and Research Committee (n° 85- 1114). The data were collected anonymously from computerized records. The patients’ informed consent was not required.

### Study population

The patients who were included had at least one positive *Pseudomonas aeruginosa* bacterial culture during their hospitalization in the department either for colonization or infection. Colonization was defined by the presence of a positive culture of the specimen and the absence of general signs of infection. An infection was defined by a positive culture, quantitatively significant, and local or general signs of infection meeting the criteria in effect [17].

The exclusion criteria were being under 18 years of age and hospitalization in the department for less than 5 days. The patients who were included were monitored until their discharge from the department.

### Clinical data collection

For each patient, the following data were collected: age, gender, SAPS II, the reason for hospitalization, mortality in the department, prior hospitalization within the 3 previous months, *Pseudomonas aeruginosa* colonization or infection, and the antibiotics received within the previous month.

Bacteriological eradication, defined as the absence of the microorganism from bacterial cultures, and clinical symptoms corresponding to the resolution of signs of sepsis related to *P. aeruginosa* infection were also noted.

### Antibiotics examined

The following antibiotics were examined:

- Amoxicillin, amoxicillin/clavulanic acid, piperacillin/tazobactam, ceftriaxone, ceftazidime, cefepime, imipenem, meropenem
- Tobramycin, amikacin
- Ciprofloxacin

For each prescribed antibiotic, the duration of treatment, the time of initiation in relation to the presence of *P. aeruginosa*, and whether or not there was an association were noted. It was also specified whether the treatment was probabilistic or documented and whether it was adapted.

### Bacterial cultures

Samples were taken by tracheal aspiration (TA) on admission to the department for patients on invasive mechanical ventilation and then repeated twice per week. Other samples were from bronchoalveolar lavages (BAL), hemocultures, or even peroperative samples, according to the context. *Pseudomonas aeruginosa* was identified by mass spectrometry (BRUCKER).

### Resistance studies

The minimum inhibitory concentrations (MICs) of the antibiotics that were examined were defined for each *P. aeruginosa* strain that was isolated, after antimicrobial susceptibility testing in a liquid medium by automated method on a Biomerieux Vitek 2 appliance. MICs were interpreted according to the CA-SFM/EUCAST recommendations that were applicable at the time of the study. The β-lactamine-resistance mechanisms and their changes in the same patient were inferred based on antimicrobial susceptibility testing when this was possible.

For *P. aeruginosa* strains resistant to ceftazidime, several complementary tests were carried out to differentiate resistance due to AmpC cephalosporinase hyper-production from that due to acquisitions by carbapenemases, extended spectrum oxacillinases (ES-OXA), or extended spectrum beta-lactamases (ESBL). These tests were:

- ✓ A comparison of the inhibition zone diameter on Mueller-Hinton agar and Mueller-Hinton agar with 2000 mg/l of added cloxacillin.
- ✓ Synergy tests on agar with cloxacillin in case of AmpC hyper-production:
  - Between ticarcillin/clavulanic acid and ceftazidime or cefepime and between imipenem and cefepime and/or ceftazidime: if positive in favor of ESBL or ES-OXA
- ✓ Identification of a metallo-beta-lactamase (MBL): by imipenem alone/imipenem + EDTA E-test.

Suspicious strains of ESBL, oxacillinase, or any other carbepenemase were sent to the National Research Center (CNR) to identify these mechanisms. Only the first mutation was taken into account in our work.

### The different resistance mechanisms

These are the acquired resistance mechanisms that were studied:

- Penicillinase acquisition (difficult to differentiate from certain resistance mechanisms by over-expression of efflux with current laboratory techniques)
- Hyper-production of inducible cephalosporinases, AmpC
- Impermeability through the loss of the porin OprD
- Over-expression of the efflux system

The mechanisms of resistance to fluoroquinolones and aminoglycosides were not examined because they were not detectable on antimicrobial susceptibility testing alone.

### Statistical study

After the first stage of descriptive statistics and verification of the distribution of values (Kolmogorov-Smirnov test), the study population was separated into 2 groups according to the occurrence or non-occurrence of mutation.

The characteristics of the patients in the different groups were compared using non-parametric tests (Mann-Whitney test) for the continuous variables due to the lack of homogeneity of the total numbers in the groups. The results are expressed in median and confidence interval at 95% (CI 95). The categorical variables for the 2 groups were compared using Fischer’s exact test. The discriminant value of the covariates of interest, according to the occurrence of mutation, was evaluated by examination of the ROC (receiver operating characteristic) curves and their associated areas under curve (AUC).

In a second step, a multivariate analysis was used to evaluate the association between the different covariates (*p* < 0.2) and the variable explained (mutation of *P. aeruginosa* strains) by the *risk ratio* measurement. On a statistical level, this information is censored. Therefore, we used a survival model, the COX model, described before [5]. After excluding the covariates with co-linearity, we used a stepwise regression (*backward elimination*) procedure by including all the selected variables then progressively eliminating those that were non-significant. Several models were tested by selecting the one for which the AUC was highest and excluding the models with an AUC < 0.8.

In the final step, a division of the population was illustrated with the use of a segmentation tree. The aim of this technique was to describe the methods of population distribution in homogeneous groups according to the existence of mutation and covariates previously selected for multidimensional analysis. We thereby tested several growth methods including the so-called CHAID (CHAID: CHi-squared Automatic Interaction Detection) method and the CRT (classification and regression tree) method, using the one for which the predicted percentages were the highest.

The analyses were done on the MedCalc® statistical software, version 15 (Mariakerke, Belgium). The segmentation tree method was carried out on the software IBM® SPSS Statistics Version 23 (Chicago, IL). *p* < 0.05 was considered to be statistically significant.

## Results

### Description of the study population

One hundred fifty-three patients were included, for whom the characteristics are indicated in Table 1. They had a median age of 64 years (95% CI 62–66). Median SAPS II was 58 (95% CI 55–63), and ICU mortality was 34%. Median length of stay was 21 days (95% CI 18–25), and the duration of mechanical ventilation was 16 days (95% CI 14–20.6).

**Table 1:** Clinical characteristics of the population and comparison of the patients with *P. aeruginosa* (P. a) without mutation *vs* with mutation

	Overall population		No mutation n= 119		Mutation n=34		*p*
	Median	95% CI	Median	95% CI	Median	95% CI	
Age (years)	64	62 - 66	65	63 - 67	61	56 - 65	0.189
SAPS II	58	55 - 63	58	56 - 63	51	41 - 67	0.356
Length of stay (days)	21	18 - 25	18	16 - 23	34	26 - 40	0.0002*
Duration of ventilation (days)	16	14 - 20.6	14.5	13 - 17	31.5	25 - 35	0.0001*
Prior hospitalization period	6	4 - 8	5	3 - 8	9	5 - 18	0.094
Colonization period	9	7 - 11	9	7 - 11	9.5	3 - 15	0.981
Infection period	10.5	7 - 13	10.5	6 - 13	10.5	2 - 15.8	0.625
Duration 1st treatment P.a 1(n: 68/30)	4	4 - 5	4.0	3 – 5.6	4.5	3 – 6	0.656
Duration 2nd treatment P.a 2(n: 41/26)	6	5 - 9	5.0	3 – 7	8.5	5.6 - 12	0.041*
Duration 3rd treatment P.a (n: 13/15)	8	2 – 10.6	6.0	1.5 – 11	9.0	2.5 - 13	0.579
Number of ATB before *P. aeruginosa* in ICU	2	1 - 2	2	1 - 2	2	1 - 2	0.598
Gender M/F			94 (79%)/25 (21%)		22 (64.7%)/12 (35.3%)		0.112
Clinical recovery no/yes	50(32.7%)/103(67.3%)		34 (28.6%) / 85 (71.4%)		16 (47.1%)/18 (52.9%)		0.061
Bacteriological recovery no/yes	121(79.1%)/32(20.9%)		89 (74.8%) / 30 (25.2%)		32 (94.1%) / 2 (5.9%)		0.016*
Death no/yes	101 (66%)/52 (34%)		79 (66.4%) / 40 (33.6%)		22 (64.7%) / 12 (35.3%)		0.840
Reason for hospitalization							
Multiple trauma	7 (4.6%)		7 (5.9%)		0 (0 %)		0.351
Medical	75 (49%)		58 (48.7%)		17 (50 %)		
Surgical	71 (46.4%)		54 (45.4%)		17 (50%)		
Sampling type							
Tracheal aspiration			77 (64.7%)		21 (61.8%)		0,698
Bronchoalveolar lavage			18 (15.1%)		6 (17.6%)		
Blood cultures			17 (14.3%)		5 (14.7%)		
Intraperitoneal sample			3 (2.5%)		2 (5.9%)		
Prior hospitalization no/yes	41 (26.8%)/112(73.2%)		33 (27.7%) / 86 (72.3%)		8 (23.5%) / 26 (76.5%)		0.826

* signify that it is considered to be statistically significant with *p* < 0.05

73.2% of the patients had been hospitalized before admission to the ICU, and 18.3% were *P. aeruginosa* carriers on admission. The “mutation” group included 34 patients and the “no mutation” group 119 patients.

### Predictive factors of mutation among the clinical characteristics of the patients

The duration of stay was significantly longer, in the univariate analysis, in the “mutation” group than in the “no mutation” group (median of 34 days versus 18 days, *p* = 0.0002), as well as the duration of mechanical ventilation (31.5 days versus 14.5 days, *p* = 0.0001). On the three lines of antibiotic treatment, the duration of the second antipyocyanic treatment was significantly longer in the “mutation” group (8.5 days versus 5 days, *p* = 0.041).

### Predictive factors of mutation among bacteriological data

Bacteriological eradication was significantly less frequent in the “mutation” group (5.9% versus 25.2%, *p* = 0.016). There was no difference in terms of clinical recovery or mortality between the 2 groups (Table 1).

In the univariate analysis, *P. aeruginosa* was significantly more often responsible for an infection than simple colonization in the “mutation” group (88.2% versus 61.3% of infection, *p* = 0.003). Initial resistance was more frequently identified in the “no mutation” group (80.7% versus 55.9%, *p* = 0.006). The type of initial resistance to the β-lactamines was distributed differently in the two groups (Table 2). Initial resistance to the aminoglycosides was only identified in the “no mutation” group (*p* = 0.001).

**Table 2:** Comparison of patients with *P. aeruginosa* without mutation *vs* with mutation Bacteriological data

	No mutation n= 119	Mutation n=34	*p*
Prior ICU colonization no/yes	98 (82.4%) / 21 (17.6%)	27 (79.4%) / 7 (20.6%)	0.801
Infection with *P. aeruginosa* no/yes	46 (38.7%) / 73 (61.3%)	4 (11.8%) / 30 (88.2%)	0.003*
Other infections no/yes	36 (30.3%) / 83 (69.7%)	5 (14.7%) / 29 (85.3%)	0.081
Initial resistance no/yes	23 (19.3%) / 96 (80.7%)	15 (44.1%) / 19 (55.9%)	0.006*
Type of initial resistance to β-lactamines			
1= impermeability	1 (0.8%)	2 (5.9%)	0.005*
3= Penicillinase and/or efflux	45 (37.8%)	11 (32.4%)	
4= AmpC over-production	10 (8.4%)	0 (0%)	
5= impermeability +/- Over-production of efflux	9 (7.6%)	4 (11.8%)	
6= 4+5	25 (21%)	1 (2.9%)	
Fluoroquinolone resistance no/yes	74 (62.2%) / 45 (37.8%)	25 (73.5%) / 9 (26.5%)	0.309
Aminoglycoside resistance no/yes	94 (79%) / 25 (21%)	34 (100%) / 0 (0%)	0.001*
Antibiotic therapy after isolation of *P. aeruginosa*			
*P. aeruginosa* ATB 1 adapted (n=97) no/yes	10 (14.9%) / 57 (85.1%)	2 (6.7%) / 28 (93.3%)	0.332
*P. aeruginosa* ATB 2 adapted (n=66) no/yes	2 (5%) / 38 (95%)	0 (0%) / 26 (100%)	0.515
*P. aeruginosa* ATB 3 adapted (n=28) no/yes	0 (0%) / 13 (100%)	2 (13.3%) / 13 (86.7%)	0.484
Tazocillin after *P. aeruginosa* no/yes	94 (79%) / 25 (21%)	23 (67.6%) / 11 (32.4%)	0.176
Ceftazidime after *P. aeruginosa* no/yes	95 (79.8%) / 24 (20.2%)	19 (55.9%) / 15 (44.1%)	0.007*
Cefepime after *P. aeruginosa* no/yes	101 (84.9%) / 18 (15.1%)	30 (88.2%) /4 (11.8%)	0.785
Meropenem after *P. aeruginosa* no/yes	79 (66.4%) / 40 (33.6%)	15 (44.1%) / 19 (55.9%)	0.027*
Fluoroquinolone after *P. aeruginosa* no/yes	115 (96.6%) / 4 (3.4%)	34 (100%) / 0 (0%)	0.576
Aminoglycosides after *P. aeruginosa* no/yes	57 (47.9%) / 62 (52.1%)	8 (23.5%) / 26 (76.5%)	0.017*

* signify that it is considered to be statistically significant with *p* < 0.05

### Predictive factors of mutation among antibiotic therapies

Of the antibiotic therapies prescribed after isolation of *P. aeruginosa*, ceftazidime, meropenem, and the aminoglycosides were prescribed significantly more often in the “mutation” group with respectively 44.1% versus 20.2% (*p* = 0.007), 55.9% versus 33.6% (*p* = 0.027), and 76.5% versus 52.1% (*p* = 0.017). Receiving antibiotic therapy adapted to the *P. aeruginosa* strain was not significantly associated with the absence of mutation.

### Predictive value of mutation among the variables of interest

The highest sensitivity and specificity as well as the negative predictive value (NPV) and positive predictive value (PPV) among the variables were those concerning the duration of ventilation. By including the influence of the duration of ventilation and of stay on the occurrence of mutation in the multivariate analysis (logistic regression), a duration of ventilation > 24 days was significant (*p* = 0.0084) while a duration of stay > 27 days was eliminated (*p* = 0.513). The initial multivariate analysis using the Cox model taking into account the thresholds of all the covariates (while eliminating those that are related) is presented in Table 3.

**Table 3:** Multivariate analysis using the Cox model

Significant covariates	Risk ratio	CI 95%	*p*
Duration of ventilation > 24 days	4.29	1.94- 9.49	0.0003*
Initial resistance	0.36	0.18- 0.71	0.0031*
Non-model variables			
Ceftazidime after *P. aeruginosa*	1.66	0.82- 3.37	0.160
Meropenem after *P. aeruginosa*	1.17	0.57- 2.40	0.674

* signify that it is considered to be statistically significant with *p* < 0.05

The AUC of the model is equal to 0.83 (CI 95% 0.758–0.884), sensitivity at 71%, and specificity at 82% (PPV at 53% and NPV at 91%). Therefore, this multidimensional analysis shows that the covariates associated with the risk of mutation are:

- A duration of ventilation > 24 h with a risk ratio of 4.29
- The existence of initial resistance with a risk ratio of 0.36.

However, for the prescription of ceftazidime after the identification of *P. aeruginosa*, the antibiotic therapy emerges from the model with a value of *p* < 0.2.

**Fig. 1:**
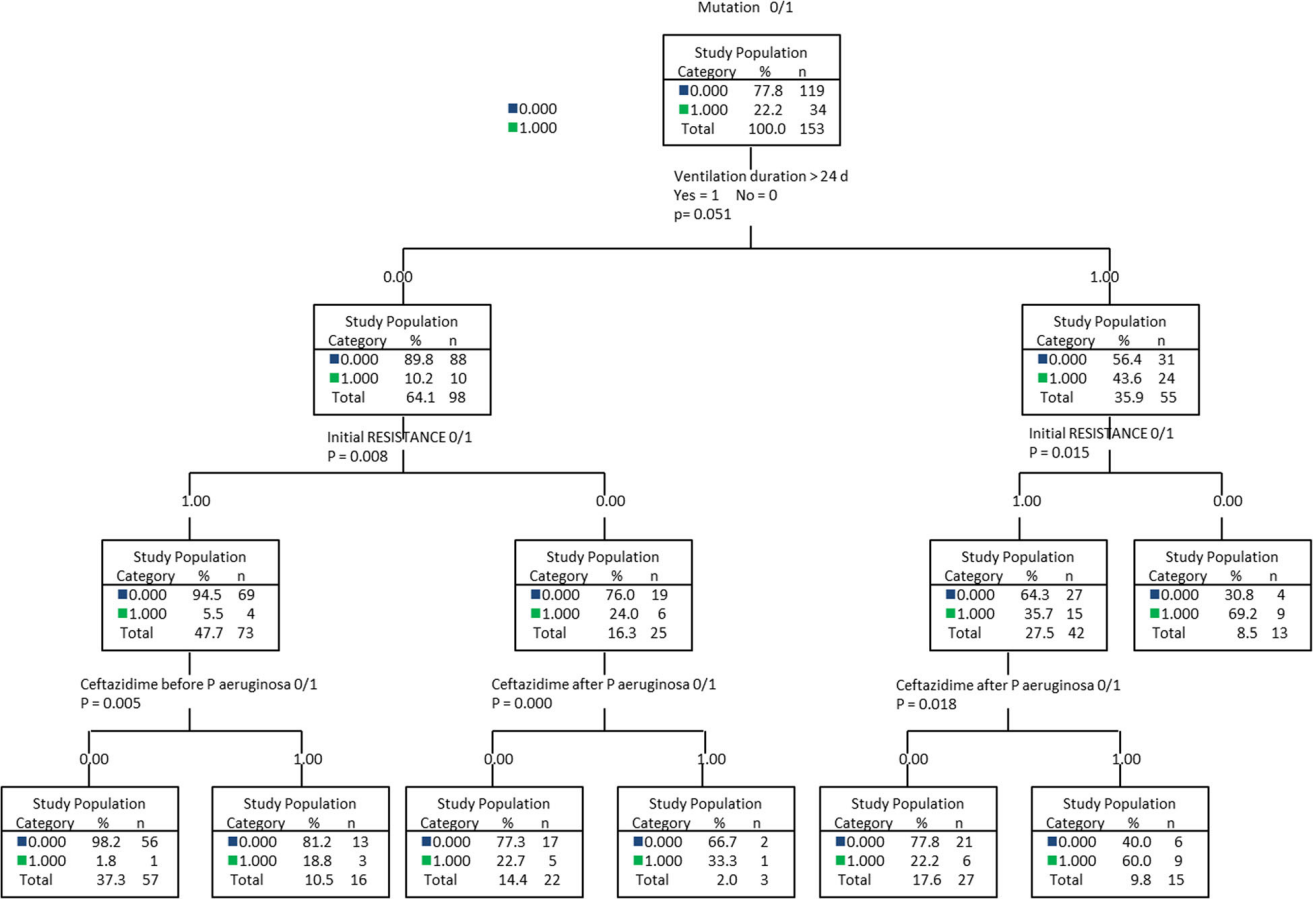
Distribution of the population according to the existence of mutation and the duration of ventilation (> or < 24 days), initial resistance, and the prescription of ceftazidime after identification of *P. aeruginosa*

The description of the methods of population distribution in homogeneous groups according to the existence of mutation and previously selected covariates (*p* < 0.2) is illustrated in Fig. 1.

The segmentation method (CRT) illustrates the dividing of individuals based on these 3 factors with a predictive value of 83%.

### Examination of the various resistance mechanisms according to the antibiotic therapies

We analyzed the relationship between the 2 antipyocyanic β-lactamines prescribed which appeared to be related to the occurrence of mutation and the different mechanisms of acquired resistance (Table 4).

**Table 4:** The relation between the 2 anti *P. aeruginosa* β-lactamines capable of generating a mutation (ceftazidime and meropenem) and the different acquired resistance mechanisms

Acquired resistance mechanisms	Ceftazidime after *P. aeruginosa*			Meropenem after *P. aeruginosa*		
	no	yes	*p*	no	yes	*p*
Impermeability no/yes	108 (94.7%) / 6 (5.3%)	38 (97.4%) / 1 (2.6%)	0.679	93 (98.9%) / 1 (1.1%)	53 (89.8%) / 6 (10.2%)	0.0135*
Over-expression of efflux no/yes	108 (94.7%) / 6 (5.3%)	37 (94.9%) / 2 (5.1%)	0.999	92 (97.9%) / 2 (2.1%)	53 (89.8%) / 6 (10.2%)	0.055
Penicillinase and/or efflux no/yes	111 (97.4%) / 3 (2.6%)	38 (97.4%) / 1 (2.6%)	0.999	92 (97.9%) / 2 (2.1%)	57 (96.6%) / 2 (3.4%)	0.640
AmpC over-expression no/yes	105 (92.1%) / 9 (7.9%)	30 (76.9%) / 9 (23.1%)	0.019*	84 (89.4%) / 10 (10.6%)	51 (86.4%) / 8 (13.6%)	0.613
Impermeability +/- Over-production of efflux no/yes	113 (99.1%) / 1 (0.9%)	37 (94.9%) / 2 (5.1%)	0.160	93 (98.9%) / 1 (1.1%)	57 (96.6%) / 2 (3.4%)	0.559

* signify that it is considered to be statistically significant with *p* < 0.05

The use of ceftazidime was significantly associated with the occurrence of an over-expression of inducible (AmpC) cephalosporinase; 23.1% of the patients who received ceftazidime developed an over-expression of cephalosporinase, compared to 7.9% of the patients who did not receive ceftazidime (*p* = 0.019). The use of meropenem was significantly associated with the occurrence of impermeability (*p* = 0.0135).

## Discussion

Our prospective study concretely demonstrates a mutation in the initial strain in 34 patients out of 153 *Pseudomonas aeruginosa* carriers during hospitalization in intensive care. The main risk factor for mutation identified is the duration of mechanical ventilation. The existence of initial resistance might protect against the risk of mutation.

Severity, mortality, the duration of ventilation and of stay, and the rate of mutation of strains in our department were similar to the previous studies on the subject [1, 18]. The rate of prior colonization and initial resistance to antibiotics are higher due to the method of recruiting patients who were often transferred to other units. In most cases, the literature is focused on identifying risk factors for the acquisition of resistant strains to a given antibiotic, especially according to prior exposure to antibiotics. We differentiated between the use of antibiotics before and during the presence of *P. aeruginosa* in order to determine the impact of exposure to the antibiotic on the microorganism and the mechanism of resistance induced.

Severity of the patients and so the durations of hospitalization and mechanical ventilation are risk factors often described as being associated with the acquisition of *P. aeruginosa* as well as resistant strains [8, 19–21]. All patients studied were severe with no significant difference between the two groups. In our study, only the duration of mechanical ventilation is significant in the multivariate analysis and seems to be the most predictive factor of the acquisition of new resistance mechanisms. The duration of any prior hospitalization and prior colonization were not identified as risk factors for mutation in our work. In most of the studies, there was a greater association between these factors and the risk of *Pseudomonas aeruginosa* infection or resistance of the strain as soon as it appears, than with the emergence of resistance [18, 22, 23].

The fact that as of its identification *P. aeruginosa* is already a carrier of resistance was noted as a protective factor against mutation. To our knowledge, this has never been described before. We also noted that the resistance mechanism that was most often acquired was an overproduction of AmpC cephalosporinase, followed by impermeability and over-expression of efflux, which corresponds with the data in the literature [3, 24]. Therefore, it would appear that a strain that is already resistant has a lower risk of developing new resistance mechanisms than a wild strain. The occurrence of a mutation was most often identified in *P. aeruginosa-*infected rather than *P. aeruginosa-*colonized patients, with less frequent bacteriological eradication. Naturally, these patients more frequently received an antipyocyanic antibiotic therapy and were therefore subject to a higher selection pressure which could explain the more frequent occurrence of mutation in these patients [25].

Most of the studies found a correlation between mortality and an infection by a resistant or even multi-resistant strain, in comparison with a sensitive strain [10, 14, 26]. However, they did not take into account the capacity of resistant strains to evolve, which our study did. Moreover, Park et al. [27] found that mortality was mainly related to the adequacy of initial treatment rather than the level of resistance of the strains. The increase in the mortality rate of patients who were carriers of resistant strains would in fact appear to be related to an adapted treatment given later than if they were carriers of a sensitive strain [5, 13]. In our study, the levels of adapted initial antibiotic therapy were relatively high. We identified a maximum of 15% of inappropriate antipyocyanic treatments in the “no mutation” group (for which the initial resistance levels were higher), compared to 25 to 35% in the literature [28].

The extent of prior antibiotic therapies is widely described in the literature [22, 23, 29]. A univariate analysis showed that of the antibiotics administrated to treat *P. aeruginosa* infections, ceftazidime, meropenem, and the aminoglycosides were significantly associated with the occurrence of mutation. This association was not identified in the multivariate analysis, possibly due to a lack of power of our study considering the low number of mutations. A recent study showed that exposure to meropenem, ceftazidime, or ciprofloxacin during the presence of *P. aeruginosa* was responsible for the emergence of resistance [11]. In compliance with the recommendations, in our study, only 4 patients received ciprofloxacin [16, 30]. In a previous study, imipenem, piperacillin-tazobactam, and cefotaxime were identified as risk factors in the emergence of resistance, but not ceftazidime [3]. However, other studies showed that ceftazidime was a risk factor for the appearance of resistance [31]. In general, the carbapenems, including imipenem and more recently meropenem, are among the antibiotics that are most often associated with the emergence of resistant strains, along with the fluoroquinolones [3, 20, 25, 30].

A factor, which might be astounding, is the frequency at which aminoglycosides are prescribed for infection. Although the multivariate analysis showed no significance in our work, some studies associated them with a risk factor for their own resistance [19, 32] or to other antibiotics [23, 33]. Piperacillin-tazobactam was not identified as a risk factor for mutation in our cohort. Its responsibility for mutation varies widely from one study to another [32, 34].

The risk of AmpC cephalosporinase induction by amoxicillin/clavulanic acid or ceftriaxone was not identified in this study [3, 35].

For acquired resistance mechanisms, our study identified a relationship between the use of ceftazidime and an over-production of inducible AmpC cephalosporinase, and between the use of meropenem and the acquisition of impermeability by loss of the porin OprD. It is responsible for the resistance attributed to imipenem, and according to the literature, it is found in 86 to 100% of the carbapenem-resistant strains [36, 37]. This acquisition, associated with exposure to the carbapenems, concerns imipenem and meropenem [19]. Several studies also describe an association between the use of ceftazidime and the over-production of inducible AmpC cephalosporinase [38]. This mechanism might explain the phenomena of co-resistance found between ceftazidime and cefepime [39], and ceftazidime and piperacillin-tazobactam [40] or of cross-resistance with other cephalosporins [41]. Exposure of *P. aeruginosa* to meropenem also tends to be associated with the acquisition of an over-expression of the efflux system.

By sending strains to the CNR (French national reference center) that were suspected of having emerging resistance mechanisms, we objectively identified a single carbapenemase carrier strain and no ESBL strain. These data are consistent with recent studies which estimate the prevalence in France of ESBL strains at 0.55% and carbapenemase carrier strains at 0.86% [24, 42].

The original nature of this work lies in the creation of a segmentation tree which enables the distribution of the study population according to the existence of mutation and covariates of interest, including a ventilation period of more than 24 days, initial resistance of the strain, and the prescription of ceftazidime after the appearance of *P. aeruginosa* with a predictive value of 83%. This tool seems useful to predict the risk of the occurrence of an event, in this case mutation, according to the variables of interest.

However, our study has several limits. When there is resistance to antipyocyanic penicillins alone, we could not differentiate the presence of penicillinase from an efflux system. It was also difficult at times to differentiate the presence or absence of over-expression of an efflux system associated with impermeability, when there was a resistance to carbapenems, if the strain also over-produced inducible AmpC cephalosporinase. The combination of an over-expression of AmpC cephalosporinase and impermeability might be enough to acknowledge that there is an associated resistance to meropenem [36–38, 43]. If such was the case, they were classified in a separate category called “impermeability±over-expression of efflux”. Therefore, the importance of an over-expression of the efflux system might be underestimated in this study. Secondly, the clonality of the different strains found in the same patient over time could not be examined, which did not make it possible to eliminate the acquisition of a new strain, if any, that might explain the change in the resistance profile. However, the literature is rather in favor of a change in the resistance of *P. aeruginosa* by mutation of the initial strain than by the acquisition of a different strain [3, 24]. When considering the segmentation tree, it does not predict a priori, from the first day of mechanical ventilation, the occurrence of a mutation. Indeed, the length of mechanical ventilation is a recognized risk factor for multidrug-resistant organisms or *Pseudomonas aeruginosa* [44].

## Conclusion

Our study confirms that the duration of mechanical ventilation of *Pseudomonas aeruginosa* carrier patients is a risk factor for strain mutation. However, the notion of resistance as soon as the strain appears is a protective factor. In a univariate analysis, of all the antibiotics examined, ceftazidime, meropenem, and the aminoglycosides seem to be risk factors for mutation. However, these results were not confirmed in a multivariate analysis. The study of resistance mechanisms acquired when ceftazidime or meropenem are prescribed has concretely demonstrated that exposure of a strain of this bacillus to ceftazidime is significantly associated with the over-expression of an inducible AmpC cephalosporinase. Exposure to meropenem is significantly associated with the acquisition of impermeability by loss of the porin OprD. For now, the emergence of carbapenemase-secreting strains or ESBL remains marginal in this population. The original nature of our work lies in the segmentation according to the predictive factors of the occurrence of resistance, i.e., the duration of artificial ventilation, the prior existence or absence of resistance, and the prescription of ceftazidime.

## Data Availability

All the samples were sent to Laboratory of Bacteriology, Federative Institute of Biology of our institution. The MICs of the antibiotics were examined in this unit. Data can be found in our intensive care unit or in the Laboratory of Bacteriology.

## Abbreviations

ATB: Antibiotic
AUIC: Area under the inhibitory curve
BAL: Bronchoalveolar lavage
CA-SFM/EUCAST: Comité de l’Antibiogramme de la Société Française de Microbiologie/European Committee on Antimicrobial Susceptibility Testing
CHU: University hospital center
CI: Confidence interval
CNR: National Research Center
ESBL: Extended spectrum β-lactamase
MIC: Minimum inhibitory concentration
NPV: Negative predictive value
P. a: *Pseudomonas aeruginosa*
*P. aeruginosa*: *Pseudomonas aeruginosa*
PPV: Positive predictive value
ROC: Receiver operating characteristic
TA: Tracheal aspiration
VAP: Ventilator-acquired pneumopathies
VS: Versus
